# A “poly-matter network” conception of biological inheritance

**DOI:** 10.1007/s10709-024-00216-1

**Published:** 2024-10-19

**Authors:** Günter A. Müller, Timo D. Müller

**Affiliations:** 1 Institute of Diabetes and Obesity (IDO), Helmholtz Diabetes Center (HDC) at Helmholtz Zentrum München, German Research Center for Environmental Health (GmbH), Ingolstädter Landstraße 1, 85764 Oberschleissheim, Germany; 2 Biology and Technology Studies Institute Munich (BITSIM), Lappenweg 16, 80939 Munich, Germany; 3 Media, Culture and Society, Department of Media Studies, Faculty of Arts and Humanities, University Paderborn, Warburger Str. 100, 33098 Paderborn, Germany; 4 Walther-Straub Institute of Pharmacology and Toxicology, Ludwig-Maximilians-University Munich, Munich, Germany

**Keywords:** Extracellular vesicles, Glycosylphosphatidylinositol-anchored proteins, Non-DNA matter, Non-genetic inheritance, Structural templating

## Abstract

Here we intend to shift the “DNA- and information-centric” conception of biological inheritance, with the accompanying exclusion of any non-DNA matter, to a “poly-matter network” framework which, in addition to DNA, considers the action of other cellular membranous constituents. These cellular structures, in particular organelles and plasma membranes, express “landscapes” of specific topologies at their surfaces, which may become altered in response to certain environmental factors. These so-called “membranous environmental landscapes” (MELs), which replicate by self-organization / autopoiesis rather than self-assembly, are transferred from donor to acceptor cells by various – vesicular and non-vesicular – mechanisms and exert novel features in the acceptor cells. The “DNA-centric” conception may be certainly explanatorily sufficient for the transfer of heritable phenotype variation to acceptor cells following the copying of DNA in donor cells and thereby for the phenomenon of biological inheritance of traits. However, it is not causally sufficient. With the observation of phenotype variation, as initially manifested during bacterial transformation, the impact of environmental factors, such as nutrition and stress, in the differential regulation of gene expression has been widely accepted and resulted in intense efforts to resolve the underlying epigenetic mechanisms. However, these are explained under a conceptual frame where the DNA (and associated proteins) are the only matter of inheritance. In contrast, it is our argumentation that inheritance can only be adequately understood as the transfer of DNA in concert with non-DNA matter in a “poly-matter network” conception. The adequate inclusion of the transfer of non-DNA matter is still a desideratum of future genetic research, which may pave the way for the experimental elucidation not only of how DNA and membrane matter act in concert to enable the inheritance of innate traits, but also whether they interact for that of acquired biological traits. Moreover, the “poly-matter network” conception may open new perspectives for an understanding of the pathogenesis of “common complex” diseases.

**Supplementary Information:**

The online version contains supplementary material available at 10.1007/s10709-024-00216-1.

## Introduction

On August 1977, at lake Schliersee, Upper Bavaria, in Germany, at a meeting on the genetics and biogenesis of mitochondria, the geneticist, microbiologist and molecular biologist Fritz Kaudewitz and the physician, biochemist and cell biologist Walter Neupert presented their differing theses about the role of DNA in the development and inheritance of cells, in general, and mitochondria, in particular. Kaudewitz defended the conception of DNA acting as the one and only matter of inheritance, i.e., as the one and only carrier of biological information of inheritance and development (for a review, see Wolf 1997). Neupert attributed to DNA a critical function “exclusively” in the time-controlled (‘one-dimensional’ and ‘linear) biosynthesis of proteins. However, and in sharp contrast to Kaudewitz, he interpreted (a subset of) organelles themselves as carriers of the information for their (‘three-dimensional’ and ‘spatial’) assembly and biogenesis, i.e., as matter of inheritance and development, in addition to (nuclear as well as mitochondrial) DNA (for a review, see Pfanner et al. 2019). That a controversial debate had happened at that meeting would not have been worth mentioning here, since this is typical for a sound scientific discourse. However, it was striking that this controversy did not sediment in a literal format in the congress booklet (Bandlow et al. 1977). Presumably, most of the meeting participants, including the editors, shared the “DNA- (environment-) centric conceptions” of inheritance. These views had already been included into textbook knowledge as the canonical conceptions at those times, as held true for the “linear, one-dimensional” interpretation of genetic information. The purpose of this perspective paper is not to neglect the merits of the “DNA- and information-centric” conception of biological inheritance with its major strength of successfully explaining and predicting the development and evolution of many phenotypic traits. This has been amply documented during the past 70 years. Rather, the proposed “poly-matter network” conception hopes to integrate additional “form and substance” into the process of matter transfer from mother to daughter cell and from one generation to the next. Consequently, the “poly-matter network” conception presented here represents an expansion rather than a narrowing or even refutation of the “DNA-and information-centric” one.

## DNA as information

During the flourishing era of modern molecular biology, the idea of the gene evolved away from chemical substance to non-material information that makes up the text of the “book of life” (Doyle 1997; Fox Keller 2000; Kay 2000; Rose 2007). This transition has started in the early 1960s and reached its (temporary) culmination at the beginning of the new millennium with the publication of the “human book of life”, i.e. the complete sequence of the human genome (Nerlich et al. 2002; International Human Genome Sequencing Consortium 2004). Around the year 2000 and during the following years, it was increasingly recognized that the metaphor of “information” was no longer appropriate and capable to cover how “life” and organisms act. Consequently, genetics and molecular biology turned their focus towards protein biochemistry, proteomics, and structural biology, as well as towards the analysis of the nature of metabolites (metabolomics) and their steady state concentrations and fluxes (fluxomics). Those disciplines emphasized the meaning of materiality of the substances involved as well as of its mutual interconversion, i.e., of three-dimensional structures, spatial relationships and molecular interactions. Concomitantly, researchers in these fields displayed less interest in information metaphors as manifested in linear sequences of letters and symbols for the characterization of DNA molecules (Jenner and Taithe 2000; Tanford and Reynolds 2001; Tyers and Mann 2003; Pappas 2006).

If one follows the historical outline of the information-driven conceptions of heritable phenotypic variation in genetics and molecular biology, it becomes obvious that a change in mindset is still underway. Changes often manifest themselves in the introduction of new metaphors. This is clearly established in genetics and molecular biology, already well developed in biochemistry, proteomics, and structural biology but only in its infancy in membrane and organelle biology. Now, times seem to be ripe for proteins, membranes, surfaces, organelles, in short “membrane landscapes” (MLs, see below for further explanations) to take over as the primary generators of metaphors and specific ways of thinking, researching, and writing about genes and DNA. Of course, this does not mean that genes and DNA will be abandoned as the “object” of scientific study. However, they may need to be examined from a new perspective, namely that of the proteome and – hopefully in near future – that of biological membranes and MLs. Under this new conceptual framework, genes and DNA will lose their meaning as the only representative and critical matter of inheritance.

It is a common place that changes in perspective and conception inevitably lead to the generation of new metaphors. In the following, we will try to show how the metaphors and narratives used in the biological inheritance discourse seem to be shifting from DNA to non-DNA matter. It is also important to note at the outcome that these shifts are not clear-cut breaks, radical transformations, or paradigmatic revolutions. But often, they emerge just as series of smaller steps that slowly undermine the previous foundation of certain metaphors, narratives and models, and pave new metaphorical and narrative paths that open up as a result of this erosion. Thus, DNA matter is not abandoned as “object” of study in the life sciences, just as “intention”, “purpose” and “meaning” have not been pushed aside in the historical sciences, but their status as the only representative and dominant generators of metaphors, narratives and models seems to be changing now.

The groundbreaking elucidation of the structure of the DNA double helix by James Watson and Francis Crick (Watson and Crick 1953) paved the path to the assumption that this macromolecule, which from a chemical point of view may be regarded as rather simple and boring, operated as the primordial substance of heredity in all living organisms. This assumption has become known among molecular biologists as the “central dogma of molecular biology” (Crick 1970). This dogma is essentially the belief that the genome of an organism, i.e., the entirety of its genes, fully explains the characteristic expression and specific combination of inherited traits, i.e., its unique “phenotype” (for comprehensive and outstanding discussions of the relationship between genotype and phenotype, see Chevin et al. 2022; de Vienne 2022; de Vienne and Capy 2022; Fisch 2022; Pontarotti et al. 2022; Robette et al. 2022; Shah 2022). It represents the foundation of a fundamental revolution that took place in molecular biology and genetics over the 20 years following the discovery of the DNA structure, which included the deciphering of the “genetic code” in 1961 (Nirenberg and Matthaei 1961). Based on this dogma, which is as simple as elegant and easy to understand, an attempt was initiated and seemingly completed to explain biological inheritance solely at the molecular level. Accordingly, the molecular substance of heredity is DNA, which is essentially a very long linear molecule consisting of only four different building blocks and is wound up in the nucleus of every cell in a strongly condensed fashion. Individual specifically “marked” sections of DNA form the genes that determine – independently or in combination with others – each of the inherited traits through a series of complex molecular processes, typically after the generation of RNA “transcripts” from the genes and then of proteins from the RNA “transcripts”.

This “DNA-centric” conception was further strengthened and expanded by a large number of important discoveries in the 1970s to up to the beginning of the next millennium. These were, in particular, the advent of recombinant DNA technology (Linn and Arber 1968; Jackson et al. 1972), the launch of the Human Genome Project (Stephens et al. 1990; Watson 1990), and the publication of the complete draft of the human genome (International Human Genome Sequencing Consortium 2004). From the 1960s onwards, these scientific discoveries led to the formulation of what is called the “information model” of life, which applies from the bacterial cell to the human body. This model was specially mediated via the metaphor of the “Book of Life”, a metaphor particularly prevalent in the rhetoric surrounding the “Human Genome Project” (Nerlich et al. 2002; Lorimer 2005; Rose 2007). Genes were conceived as a kind of digital manual for the creation of all organisms, including humans. While DNA itself is matter, i.e., a physical substance of spatial structure and specific materiality, the ways in which DNA was assumed to be responsible mainly or even exclusively in generating the phenotype has been shrouded in metaphors and narratives of code, semiotics, text, letters, errors, writing, reading and erasing. This view of the genome as an information system, as a linguistic text written in the DNA code, has guided the theories and practices of molecular biologists since the 1950s (Brandt 2005). It culminated in deciphering the “Book of Life”, a narrative that has developed a life of its own with great impact (Kay 2000, p. 325). This view was also manifested in a speech by former US President Bill Clinton on June 26, 2000, at the press conference announcing the publication of the working draft of the human genome.

The information model promises people the opportunity to read the “Book of Life”, an “opus” that until now has only been accessible and understandable to its writer or creator. And accessible not only to read, but also to rewrite it, as Hans-Jörg Rheinberger (2000) pointed to the fundamental change that recombinant DNA technologies have brought about. According to this conception, life is something that is “written” and “read”. It is basically a linear text, and it is a text that scientists can not only read but can also edit and rewrite. Even without a further outline of how complicated this conception ultimately is, and despite the shift of the focus in genetics and molecular biology to biochemistry and proteomics, the importance of the information model in efforts to identify the basis of inheritance for all vital processes, from normal function, physiology, and behavior to dysfunction, aging, and disease, cannot be overestimated. The information model still has significant narrative and metaphorical impact, as well as economic, political, and cultural influence. And no doubt, as one of its major merits, it has provided a consolidated conception about nature, life, heredity and organisms in the public and published opinion (Haraway 1997; Weber 2003; Rose 2007), to up to the point that it has created a kind of “genetic fetishism” (Kay 2000, p. 342).

### The “DNA- / environment-centric” conception of inheritance

The central concept of an epistemology of the information model of inheritance is the so-called “gene-for-thinking”, i.e., the search for the one and only gene that codes for the corresponding different manifestations of life or for the specific differences between organisms, being it specific functions, unique for certain but no other organisms, such as production of a venom, different phenotypic characteristics, such as eye color or body size, or different (patho)physiological configurations, such as a special mental or physical performance or a disease. This way of thinking with its oppressive narrowness becomes manifested in the countless studies on the search for *the* musicality gene, *the* intelligence gene, *the* obesity gene, *the* alcoholism gene, *the* homosexuality gene, etc. While some scholars warned from the beginning against believing this rhetoric, the public imagination and not only that held on to it. But as genetics and molecular biology has evolved, an increasingly influential group of scientists hinted to the limitations of the information model. It has been increasingly recognized that, on the one hand, many genes do not code for a single protein or a protein at all and, on the other hand, many (patho)physiological processes are determined by a multitude of interacting proteins, i.e., by a complex network of proteins, rather than a single protein.

Despite this, or eventually because of this, genetic sequence data are still growing exponentially across all organisms, from bacteria to humans. Google’s massive investment in the personalized genetic data service “*23andme.com*” is a perfect example of the scientific, economic, but also emotional significance of such data for people. Scientists repeatedly emphasized that these versions, or rather versions of genomes from different organisms, are based on a simplified and reductionist understanding of the gene. Such sequence data seem to contain more data than they actually do and promise existential meaningfulness and impact that they do not and cannot provide. The transformation of DNA sequences into products, i.e., transcription, translation, post-translational modification, subcellular targeting, is controlled by many intrinsic and extrinsic factors and is also extremely context-dependent, i.e., is decisively influenced by the neighboring sequences. This contradicts DNA sequencing’s belief in the explanatory power of genomic sequences, a belief based on a relatively simple match between genes, structures, and functions. The experimental evidence is overwhelming that a simple direct relationship between genotype and phenotype does exist only in exceptional cases. Over the past five decades, life has been transformed from the simple deciphering of a molecular (i.e., genetic) code to the mysterious transformation of the one-dimensional, linear, and comparatively simply structured genome into the three-dimensional networking and very complex system of proteins, membranes, subcellular structures, cells, organisms and bodies. Nevertheless, the following groundbreaking experimental findings and milestones in biochemistry, microbiology, genetics and molecular biology, starting in the 1950s and often awarded with the Nobel Prize, have initially been interpreted under a view which adhered to the information model:

(i) In the early 1960s, the Danish biochemist Christian Anfinsen described the successful re-folding and restoration of the hydrolysis activity of the enzyme ribonuclease following its complete denaturation and unfolding and the resulting total loss of a defined three-dimensional structure and its enzymic activity (Anfinsen and Haber 1961).
(ii) In the early 1960s, the American biochemist DLD Caspar and Aaron Klug (1962) together with the virologists Heinz Fraenkel-Conrat and Robley Williams (1955) demonstrated the reconstitution of infectious tobacco mosaic virus particles in vitro from their purified constituents, the capsid protein that constitutes the viral envelope and the single-stranded RNA trapped within the envelope. This process has been called “self-assembly” because no molecules are involved and required that are not themselves components of the newly assembled functional biological structure. Thus, the viral capsid protein and RNA are necessary and adequate (i.e. causally sufficient, see below) for assembly of the infectious tobacco mosaic virus particle.
(iii) Subsequently, the principle of “self-assembly” was demonstrated for the “spontaneous” formation of a number of protein-nucleic acid complexes, including the heads of DNA bacteriophages (King et al. 1973; van Driel 1997; Rossmann et al. 2004), the subunits of ribosomes (Venema and Tollervey 1999), as well as specific protein-ribonucleic acid complexes, such as the signal sequence recognition particle (Massenet 2019). In some of these cases, however, the functional assembly was crucially dependent on the proportion of the individual constituents and / or their emergence along a defined temporal sequence and spatial arrangement and / or on the support of “scaffolding proteins”, such as chaperons, which are not contained in the final functional structure (Seth Horne and Grossmann 2020).
(iv) In the 1950s, Robert Briggs and Thomas J. King (1960) succeeded in the first transfer of isolated nuclei (from skin fibroblasts) into embryonic cells (“enucleated” oocytes of the frog *Xenopus laevis*). Subsequently, in the 1960s, John Gurdon and coworkers (Gurdon et al. 1958) were able to demonstrate the complete reprogramming of the transplanted cell nuclei (from intestinal epithelial cells) upon transplantation into “enucleated” oocytes of *Xenopus laevis*. Thus, pluripotent stem cells (PSCs) were apparently obtained from differentiated cells which managed to differentiate into all cell types of an adult organism (Gurdon and Laskey 1970). During subsequent decades this pluripotency was demonstrated for mammalian organisms, including mice (Hochedlinger and Jaenisch 2002) and sheep (Wilmut et al. 1997).

Under the impression of the still overwhelming genetic information model, those data were interpreted in perfect agreement with it on the following basis: The information for (i) the three-dimensionality of proteins (i.e., their tertiary structure), as well as for (ii) and (iii) the assembly of (multi-subunit) protein complexes or protein-nucleic acid complexes exhibiting a defined quaternary structure, and for (iv) the ontogeny of organisms are all ultimately determined by the structure and function of polypeptide chains which is critically and exclusively determined by the linear amino acid sequences, with the “primary” information for the protein structure and function solely encoded in the corresponding genes. What else should be required for the development of all the structures and functions in cells, organisms and even the human body, with its huge variety? The consideration of the involvement of non-DNA non-informational factors seemed to be superfluous, even if one takes into account biological membranes, cell surfaces, and skin for the demarcation of cell and organismal boundaries as well as the separation of interior and exterior environments.

However, along this period some system theorists have expressed great reservations about this interpretation of genetic information (for instance see Gray 1992; Oyama 2000; Griffiths 2001). They argued that such a distinction between genetic informational reasons and non-DNA non-informational reasons was not empirically justified and was founded on incorrect metaphysical assumptions. Moreover, the use of the metaphor “genetic information” has been criticized by numerous geneticists as well as philosophers of science (Fox Keller 2000; Nelkin 2001; Venville et al. 2006; Walsh 2020). This critique of the concept of “genetic information” has in turn been characterized by others as – at least in part – exaggerated (Wheeler and Clark 1999; Maynard Smith 2000; Sterelny 2000; Kitcher 2001). A deeper discussion into this complex discourse is not necessary at this point. Simply, from the assumption that the genetic causes of development (of heritable traits and phenotypes) are informational, it cannot be concluded that genetic causes are explanatorily sufficient and that non-DNA causes are to be interpreted only as an explanatory background. If somebody wants to explain the characteristics of a roast, the reference to the recipe, i.e., the relevant information used is usually not sufficient. A better explanation would be to indicate which ingredients have been used and in the quality of the ingredients. In addition, it should also be stated which cooking and frying utensils and equipment have been applied and eventually how they have been operated. And of no minor importance would be mentioning who the cook has been and what training, qualifications, or competencies he or she had. A roast cooked from low-quality products or by incompetent people using inappropriate utensils will typically not be of high taste, regardless of the type of or adherence to a recipe that has been put together per se for a good roast. Recipes are usually not sufficient to explain the characteristics of a roast. So why should “genetic recipes” be sufficient to explain “phenotypic cooking”? The metaphor of “genetic information” is not sufficient per se to justify the view that all non-DNA non-informational factors (materials as well as environmental conditions) in the development of phenotypic traits are merely explanatory background, as Wheeler and Clark (1999) have claimed.

In this context, it may be instructive to point out that Gregory Bateson (1976) introduced the metaphor of the phenotype as a cake and development as cooking. He used these metaphors to argue that (i) phenotypes arise as a result of complex interactions between genetic and non-genetic “ingredients”, (ii) genes are not explanatorily privileged, and (iii) quantifications of the causal contributions of genetic and non-DNA factors are not possible or useless. Are there any other justifications for the view that non-DNA, non-informational factors are not necessary in terms of an explanation for inheritance and development? In fact, one is to consider that only the genetic, DNA, informational ones are subject to natural selection among all the factors responsible for development and inheritance. Numerous, sometimes divergent arguments have already been put forward against this view that only genetic factors become exposed to natural selection (Griffiths and Gray 2001; Sterelny 2001). But even if only genetic factors were the target of natural selection, it would not “automatically” follow that non-DNA factors should not be explicitly included in explanations of the development of inherited phenotypic traits. Rather, development may be the consequence of the causal interaction between genetically selected and non-DNA non-selected factors. The motivation of many biologists to treat non-DNA actors as an explanatory background in the case of “like-from-like” phenomena (Müller and Müller, manuscript submitted) may be due to the “claim” that the explanatory foreground has to be engaged in the generation of specificity during development. Although both genetic and non-DNA actors are generally considered to play a causal and necessary role in developmental processes, only the former are considered to contribute to specificity (Raff and Kaufmann 1991). However, it should be stated that the use of the term “specificity” in connection with developmental processes and genetic information by biologists is more likely to be assigned to the realm of intuition.

Regarding the inheritance of biological traits, the distinction between a sufficient explanation, presented in the foreground, and a sufficient causality of factors, only sometimes given in the foreground but usually hidden in the background, implies that the causal effect of some of these factors is simply self-evident, since it is generally accepted, especially by the “scientific community” as canonical knowledge. Thus, the causal involvement of those factors no longer appears to be worth mentioning, whereas that of others does. The latter apparently holds true for DNA and genes, which are then “consistently” classified as explanatorily (if not causally) sufficient. By contrast, the former applies to non-DNA matter, such as proteins and membranes, since those are typically evaluated as neither explanatorily nor causally sufficient, but as causally necessary or simply self-evident .

**Fig. 1:**
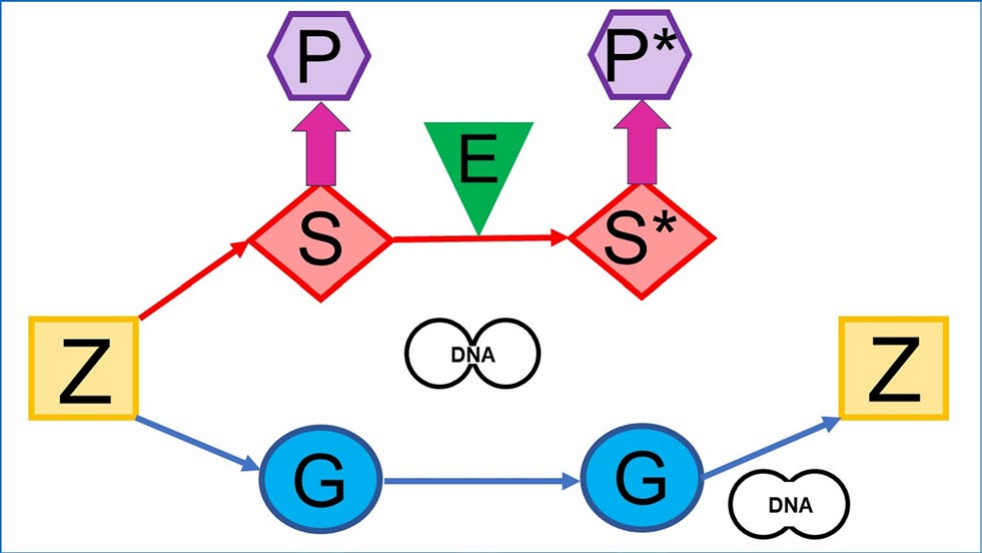
“DNA- / environment-centric” conception. During early development of the zygote (Z), produced by the parents, the somatic (S) and germ cell (G) lineages become totally separated. The entirety of S, which are copied by cell division and then become differentiated, creates the phenotype (P) of the organism. G which is also copied by cell division during adult life may ultimately lead to Z of the next generation upon cell fusion which will develop in the absence of any direct effect exerted by S. According to the “DNA-centric” conception, during the course of both cell division and fusion, DNA is the only matter to be transferred from the mother to daughter S and G, as well as from the parental G to the offspring Z, respectively. The “DNA-centric” conception has been supplemented with the impact of environmental factors (E) which induce specific changes on S, such as mutations, leading to differently differentiated and functional S* and creating an altered P*. DNA is thought to be the only matter to be transferred from S to S*, mother to daughter G and parental G to offspring Z along cell division and cell fusion, respectively, and to remain unaffected by E

It is noteworthy that over the past five decades – in addition to genes and depending on the inherited phenotypic trait – environmental conditions have successfully “struggled” for acknowledgment as factors of explanatory (and causal) adequacy for the development of phenotypes by both scientists and laymen. Accordingly, the majority – but not the entirety – of the heritable phenotypic traits and the differences between organisms are thought to be determined by the interaction of genes / DNA and environmental factors. Strikingly, this canonical DNA- / environment conception of inheritance (Fig. 1) continues to systematically exclude other factors, namely non-DNA materials, among them proteins and membranes and other subcellular structures and assemblies.

On the one hand, the “DNA- / environment-centric” canonical conception presents genes and environmental factors as explanatorily sufficient and causally necessary for the inheritance of phenotypic traits. On the other hand, proteins, membranes and others non-DNA cellular elements are presented only as explanatory background. How did this unjustified differentiation between explanatory foreground and background, causal sufficiency and necessity of DNA, environment and non-DNA elements come about? The description of the forces and motivations at the various societal, cultural, and economic levels underlying this obvious trajectory of the “DNA- / environment conception” of biological inheritance should be at the center of future Science and Technology Studies.

### The “DNA- / epigenetics-centric” conception of inheritance

In epigenetic studies, in general, and epigenetic-environmental studies, in particular, dualisms and binary differentiations, such as organisms and environment or nature and nurture, are essentially dealt with. At the same time entanglements, indeterminacies and simultaneities become apparent when, for example, epigenetics is conceived as a “mediator” and as the “intermediary” or “in-between” genes and the environment (Leuzinger-Bohleber and Fischmann 2014; Subramanaiam 2014). The question therefore arises as to what the implications are when binary boundaries dissolve and shift. Does this possibly lead to something other than just a binary understanding of nature-culture, body-mind, gene-environment, body-surroundings, as materialist and feminist philosophers of science have repeatedly suggested? (Weber 2003). The problem with those binaries is that it typically remains unclear where the boundary between the apparently antagonistic entities is positioned. Regarding the gene-environment dichotomy, where does the action of genes, e.g., responsible for melanin production, become initiated and where does the effect of UV-light end, and vice versa. Putative answers are “at the level of the gene”, induction of melanin expression in melanocytes, synthesis of melanin protein, its transport to the cell surface, distribution to keratinocytes, protection against UV-light due to conversion into heat, and “at the level of UV-light”, its generation by the sun, propagation *via* the atmosphere, partial absorption by sun crème and clothes, stimulation of the synthesis of melanocyte-stimulating hormone, and induction of melanin expression. Thus, it is simply not feasible to exactly define the terms “gene” and “environment” and to simply delineate the boundary between them, as it is true for most binary systems.

Conrad Hal Waddington is usually credited as the one who introduced epigenetics into the areas of embryology and genetics in the 1940s (Squire 2017). The term is considered a neologism from “genetics” and “epigenesis” (Müller and Olsson 2003). It resulted from debates about whether preformation or epigenesis, respectively, provides the correct view for the development of organisms. From the perspective of preformation, developmental processes could be explained from what was already there before. New things, such as molecules, structures, organs, would develop from existing ones. This was based on the early idea that the complete organism is already contained in the egg or sperm in miniature form and the embryological development is based solely on its growth (Müller-Wille 2014). While preformation is associated with a more deterministic and reductionistic explanatory model, “epigenesis” is considered holistic because it focuses on the adaptability of organisms (Schuol 2016). “Epigenesis” thus focuses less on the pre-existing than on the ever-new emergencies in the embryological course of development as well as the interaction with environmental factors. Waddington drew on both perspectives. He developed an epigenetic understanding of development in concert with an explicitly non-preformistic genetics. Consequently, he emphasized the importance of genes as well as described environmental factors as one aspect among many factors influencing developmental trajectories (Waddington 1957).

Of particular importance is the concept of “epigenetic landscape” introduced by Waddington (1940), which refers to the process of ”decision-making” of cells or tissues for a developmental pathway. This represents a surface embedded in a multidimensional space of cellular metabolism (Slack 2002). There are also illustrations that show the underside of the epigenetic landscape. There, the supporting pegs of the surface represent genes that together form and shape the landscape. Depending on the nature of the landscape, a sphere moves on it and can take different paths until it reaches its destination, the fully differentiated tissue (Waddington 1940).

For Waddington, the ability of cells and tissues to respond to an impulse or signal from the environment, as well as the “canalization” of development through numerous branched decisions are still under the control of genes (Slack 2002). Waddington was less interested in the specific triggering signals (‘inducer’), but above all in the fact that cells react or can respond differently to signals, making different developmental pathways possible (Waddington 1968). The epigenetic landscape stands then as a dynamic system in which cells and tissues react differently to signals, taking multiple developmental paths in different regions. At the same time, there is no arbitrary development but a limited number of possibilities, and everything remains genetically controlled. Depending on the level of development of the organism and action of environmental stimuli, genes shape the epigenetic landscape. Thus, what happens is not genetically predetermined. Consequently, a hard gene determinism or “DNA-centric” conception seems to soften. It becomes clear that Waddington opened up to the complex of environmental influences. At the same time, he adhered to the idea of a program according to which development takes place and does not completely neglect the conception of the gene as determinant. With the concept of canalization, Waddington made it clear that the course of development depends on numerous factors and that evolution selects canalized developmental trajectories, i.e., epigenetic trajectories that show some resistance to being changed, which are also genetically controlled. However, since development is constantly being redefined, it is nevertheless not predetermined. In our view, although Waddington acknowledged influencing factors and environmental influences, he remained within a “DNA-centric” conception and his model of ’canalization is therefore a “DNA-centric” one. In addition, Waddington’s ideas expanded the concept of heredity, as he stated: *We need a heredity system which does not merely contain information*,* but which acts as algorithms or programmes and thus leads to the production of a phenotype which takes its place between the genotype and the environment. It is the phenotype which acts on the environment (for example*,* in metabolism) and it is on phenotypes that the environment exerts its natural selective forces.* (Waddington 1968).

Waddington also used the term “program”, which assumes a fixed sequence of developmental steps (not to be mixed up with “open programs”, which may result in different phenotypes under different environments). At the same time, he emphasized the interactions between genotype and environment, which he described as nonlinear, but ongoing in different directions. A famous example of this interplay is the formation of skin calluses on the chest of ostriches. Waddington assumed that calluses had developed on the chest of the ostrich ancestors in response to environmental factors in the developmental system. Since it proved to be an advantage to sit on hot and rough ground, these have been preserved (Waddington 1942). This implies something that is still characteristic of epigenetics today: Environmental factors have led to the formation of skin calluses during life, and the descendants were born with these, although they have not longer been exposed to those environmental factors. It is common knowledge that most, if not all, organisms share the capability to modify their specific characteristics in direct response to altered environmental cues. This phenomenon has been termed phenotypic plasticity. Waddington’s explanation for this was what he called “genetic assimilation”, the process “by which a phenotypic character, which initially is produced only in response to some environmental influence, becomes, through a process of selection, taken over by the genotype, so that it is formed even in the absence of the environmental influence” (Waddington 1961). Waddington recognized that a specific phenotype which had been triggered by a given environmental cue may acquire a constitutive nature, and be maintained during normal environmental conditions, even after termination of the initial environmental cue, provided selection for organisms had continued for several generations which display highest susceptibility for expressing this phenotype upon stimulation. He correctly argued for an increase in allele frequency as consequence of the selection leading to improved, i.e., more reliable and consistent expression of the altered phenotype (Waddington 1953). This hypothesis has further been supported in the course of experiments preformed with inbred stocks missing genetic variation which were resistant toward genetic assimilation in response to selection (Scharloo 1991).

In this sense, phenotypic plasticity is lost, i.e., development is canalized, through natural selection, which drives the fixation of genetic factors that were either already present, but cryptically and in low frequency in the original population, or produced by *de-novo* mutations. In other words, driven by the process of “genetic assimilation” selection apparently manages to abrogate this environmental sensitivity, leading to fixation of a previously environmentally provoked trait. Thus, “genetic assimilation” may be attributed a key role in the emergence of novel phenotypes and even the origination of new species. The identification and characterization of the mechanisms underlying phenotypic plasticity and genetic assimilation are suggested to advance our understanding about innovation and diversification during evolution (for a review, see Pigliucci et al. 2006; Ehrenreich and Pfennig 2016; Futuyma et al. 2014; Kasinathan et al. 2017).

In his texts, the term ‘response’ is of central importance, as it is used to negotiate the relationship between gene-environment and nature-culture. ‘Responding’ leads to two other essential ingredients in Waddington’s work: genetic assimilation and the inheritance of acquired traits, are ideas still heavily debated within the field of epigenetics. Through the concept of canalization and genetic assimilation, Waddington demonstrated that environmentally induced phenotypes acquired over the course of a lifetime may become heritable. This ties in with the evolutionary ideas formulated by Jean-Baptiste de Lamarck. Lamarck assumed that traits and characteristics acquired during the lifetime of an organism can also be inherited. Although Waddington’s ideas may apparently be Lamarckian in some sense, his concepts of canalization and genetic assimilation were developed under the frame of the evolutionary synthesis, and thus can be considered within the DNA-centric conceptual model of inheritance. However, it is clear that Waddington emphasized the relevance of environmental factors for the course of development and for heredity (Waddington 1940): *From each phenotype you have to map back to a genotype*,* passing through a space of ‘epigenetic operators’which is not wholly constituted by the active genes*, *but in which environmental influences may act as programme modifiers* (the underline is ours). However, as it has been well recognized since then, the mapping of the genotype-phenotype correspondence is not essentially one-to-one.

Importantly, two different evolution theories which are both based on phenotypic plasticity have been developed, one by James Mark Baldwin at the end of the 19th century (Baldwin 1896) and some decades later Waddington, which subsequently have often been mixed up with one another. Baldwin interpreted phenotypic plasticity in the sense of the survival of an individual organism under given novel environmental conditions which thereby will affect its future evolutionary adaptations (Baldwin 1902). Differences between organisms which can be inherited are then selectable as a consequence of direct evolutionary alterations of the phenotype. Operation of this putative mechanism has been acknowledged as the “Baldwin effect”. In contrast, genetic assimilation as introduced by Waddington has to be understood as a mechanism through which a phenotype produced by a certain environmental condition, i.e., an acquired trait, undergoes canalization provoked by selective pressure operating at the developmental system. West-Eberhard (2003) and shortly thereafter Crispo (2007) introduced the term “genetic accommodation” for the causal relationship between the production of heritable alterations and the emergence of novel triggers. Accordingly, genetic accommodation seems to be instrumental for an understanding of the “Baldwin effect”. Interestingly, despite the obvious differences between genetic assimilation and the “Baldwin effect”, both mechanisms may be understood as a specific type of genetic accommodation (Crispo 2007) which could critically contribute to diversification during evolution.

The core of West-Eberhard’s and Crispo’s controversial hypothesis of genetic accommodation seems to be based on more extensive genetic shaping of features compared to mere genetic assimilation and thereby reflects the so-called Baldwin effect, as least as understood by Simpson (1953). According to him, the Baldwin effect is compatible with the common theory of evolution by natural selection, albeit he was aware of the observation of only a very limited number of natural examples so far, and doubted their broad realization, in agreement with the current state of observational studies. Following these lines, West-Eberhard argued for (i) initial induction of a phenotypic alteration to achieve adaptation to environmental changes which results in increased fitness, i.e., acquisition of phenotypic plasticity, and (ii) subsequent modification of allele frequencies, that finally leads to fixation and possibly fine-tuning of the acquired novel feature, i.e. genetic assimilation, resulting in the stabilization of a new species-specific trait (West-Eberhard 2003, pp. 157–158): *Most phenotypic evolution begins with environmentally initiated phenotype change…The leading event is a phenotypic change with particular*,* sometimes extensive*,* effects on development. Gene-frequency change follows*,* as a response to the developmental change. In this framework*,* most adaptive evolution is accommodation of developmental-phenotypic change. Genes are followers*,* not necessarily leaders*,* in phenotypic evolution.* During the last two decades this provocative speculation has produced considerable interest in the identification of development as one of the most relevant factors which fosters or even drives evolution (Gilbert and Epel 2009).

According to Gilbert (2012), the idea that interactions with the environment are relevant for development already existed in embryology in the late 19th century. Waddington was one of those to take this up again. With the advent of the concept of epigenetics in the 1940s in Europe and North America, the influence of environmental factors on the development and transmission of traits acquired over the course of a lifetime was again discussed. For this reason, environmental epigenetics has enjoyed great attention since then. What does the opening to the environment mean for the differentiations between gene-environment, nature-culture / nurture, inside-outside of cells, organisms, bodies? Are there conditions conceivable that make it possible not to think in those binary categories or dualistic paths? This development towards a strengthening of gene determinism – or ‘gene fetishism’ as Donna Haraway called it (Haraway 1995) – continued until the end of the 20th century (for instance, see Weber 2003) and can also be observed in current scientific debates (Müller-Wille and Rheinberger 2009).

Waddington’s work is characterized by a distancing from gene-deterministic explanatory models, as he emphasized the influence of and interplay with environmental factors (Waddington 1957). Thereby, a more complex and dynamic picture of embryonic development and inheritance was drawn, which at the same time argued at the logical level through monocausal determinism. This oscillation can also be found in today’s epigenetics. Schmidt (2014) also notes that Waddington thought in ‘DNA-centric’ terms, describing genes as regulating, but that he was open to other developmental factors and all the processes operating between genotype and phenotype. Unlike today’s epigenetics, Schmidt (2014) suggests that Waddington did not focus solely on gene regulation and DNA-associated molecular processes. The question of what is positioned “in between” – between genotype and phenotype or between genes and environment – is a very important starting point for dealing with differentiations and exclusions. Even in current epigenetic research, the position “in-between” continues to be negotiated.

**Fig. 2:**
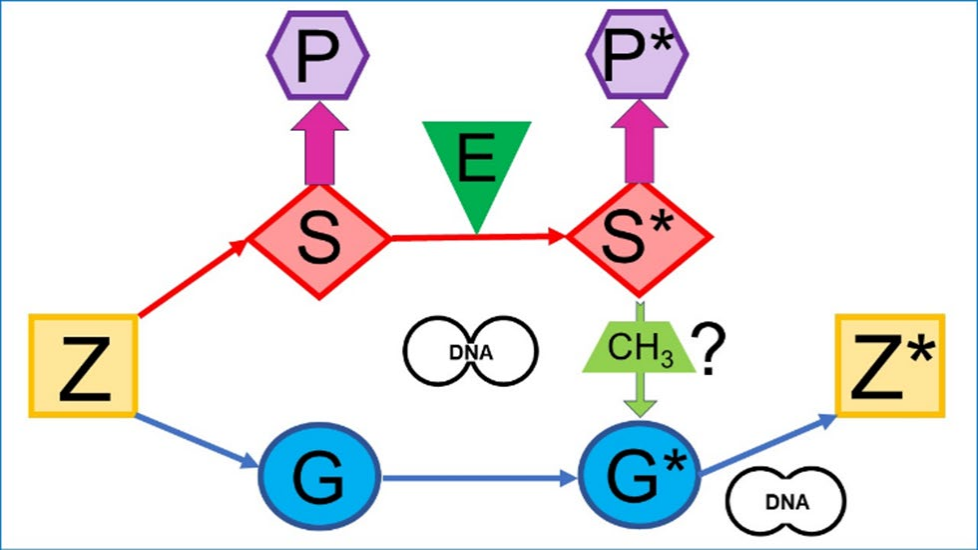
“DNA- / epigenetics- / environment-centric” conception. The “DNA- / environment-centric” conception (Fig. 1) was supplemented with epigenetic mechanisms operating at the DNA of G* because of E which induce specific changes on S*. The resulting change in P* will be inherited to the next generation in course of development of Z*. DNA is thought to be the only matter to be transferred from S to S*, G to G* and parental G* to offspring Z* along cell division and cell fusion, respectively, and to become modified by E through methylation or acetylation of itself or acetylation of associated (histone) proteins

Epigenetics should overtake the role of the “in-between”. But this forming of a bridge between the nature or the ‘given’ and the nurture or the environment goes far beyond the mere analysis of switching genes on and off through imprinting at their promoter, i.e., the addition or removal of methyl groups at cytosine-guanosine base pairs of the DNA or post-translational modifications of DNA-associated (histone) proteins. The position “in-between” should become characteristic of that research area and include efforts to determine the interactions of gene-environment, nature-culture / nurture, inside-outside under a “DNA- / epigenetics / environment-centric” (Fig. 2) rather than a mere “DNA-centric” or “DNA- / environment-centric conception (Fig. 1). One formidable approach regarding the dualism of body and cognition has recently been presented by Meloni and Reynolds (2021). They managed the rethinking of the relationship between embodied cognition and (epi)genetics, the latter so far apparently predominantly supporting the enactivistic view of mind and life.

Despite the focus on DNA of the current epigenetic research, the exact mechanisms of replication and the chemical nature of the triggers of these DNA modifications have remained unclear so far. In this context, the terms “epigenetic programming” (Block and El-Osta 2017; Alyamani and Murgatroyd 2018) and “cell memory” (Jablonka and Lamb 1998), which are passed on to the new cells, are concepts commonly used. “Memory” serves as an explanation for the fact that cells and tissues at the same location always develop in the same way and differentiate into the same cell or organ type, even without the influencing factors that guided their ancestor cells. Thereby, despite their pluripotency, a certain stability has to be explained as has been stressed by Godfrey and coworkers (2013): *Epigenetic changes*,* in particular DNA methylation*,* provide a ‘memory’ of developmental plastic responses to early environment and are central to the generation of phenotypes and their stability through the life course.* However, again in most cases it is still unclear how these epigenetic imprints and memories become transferred and inherited. Nevertheless, at the same time it is discussed that erasing of the epigenetic markers may take place when new cells and especially new generations of organisms are produced. It remains to be clarified whether everything will be deleted, i.e., whether epigenetic modifications are completely reversible. Eva Jablonka and Gal Raz (Jablonka and Raz 2009) rather assume “*an intermediate between the two extreme poles of complete reset and faithful reconstruction*.”

The different assumptions about the inheritance of epigenetic modifications have led to intense scientific controversies. Research on this has so far been very rudimentary and is mainly discussed in relation to “genomic imprinting” as an epigenetic inheritance process (Wossidlo 2012). Here “genomic imprinting” refers to the “imprinted” genes that can be inactivated by methylation and thereby be prevented from their transcription (Bajrami and Spiroski 2016). Tollefsbol (2011) postulated: *It is now apparent that epigenetic processes can be transferred from one generation to another*. A closer analysis of the data, however, shows that such a consensus is by no means given in this area of research. Rather, only the intragenerational and cellular transfer of epigenetic markers is undisputed, but not the transfer of altered gene activity patterns to the next generation and the offspring. This reveals a much larger scientific debate as Schmidt (2014) stated: *Most controversial is the significance of transgenerational epigenetic inheritance for evolutionary processes and the question of whether environmentally-induced epigenetic changes that an organism acquires in the course of its life are hereditary in the full sense – i.e.*,* whether one should actually speak of transgenerational epigenetic inheritance.* Schuol (2014) distinguishes between heredity and transgenerationality, defining inheritance as the transgenerationally stable, i.e., continuous, transmission of material information carriers by means of the germline, and considering transgenerational effects as not necessarily implicit for inheritance. Toepfer (2014) also proposes to differentiate between heredity and transmission. And Lux (2015) calls for at least a careful use of the terms “heredity” and “transgenerationality” in epigenetics.

In conclusion, epigenetic research remains to be critically evaluated in terms of its reductionistic, deterministic or “DNA- (environment-) centric” conceptions. Many researchers in epigenetics believe that it is worth taking a closer look and finding out which non-reductionistic / deterministic or pluralistic / holistic perspectives are contained in it and working them out accordingly. It is in this sense that Robert Lickliter and David C. Witherington (Lickliter and Witherington 2017) may be interpreted when they write: *Epigenetic processes are emergent properties of historical and situated relations across multiple levels of biological organization. This inclusive perspective on epigenetics provides a framework to describe and analyze dynamic processes at many levels of organization*,* without an implicit bias about what factors / parts of the system are driving or controlling the process.* In any case, so far epigenetics continues, at varying degrees, to propagate the excluding “DNA- (environment-) centric” conception of inheritance insofar it solely acknowledges the transfer of modifications of nucleotides and DNA-associated (histone) proteins (at promoter and regulatory sequences) as the communication of environmentally controlled quantitative information for the synthesis of proteins at the correct time point, exclusively.

### “Membrane landscapes” and the “poly-matter network” conception

What properties should hereditary, inherited, or heritable matter have? On the basis of adapting the metaphor of biological inheritance from the societal-cultural sphere (see Müller 2024a, b), any material that (i) becomes transferred from mother to daughter cells (asexual reproduction by division of unicellular organisms or somatic cells) or from parents to their offspring (through sexual reproduction by fusion of gametes) and that (ii) is causally involved in the development of the specificity of the acceptor organisms (daughter cells, offspring) can be understood as matter of inheritance. In addition, and restricted to biological inheritance, the ability of replication is regarded as criterion (iii). Various cellular structures and assemblies, among them plasma membranes (PMs), organelles and the MLs fulfill the criteria (i-iii) of this “operational” definition of the matter of biological inheritance. In particular, PMs are specific assemblies of proteins (including transmembrane, peripheral proteins, cytoskeletal, lipid-modified, and glycosylphosphatidylinositol-anchored proteins) and (glyco)phospholipids in bilayer configuration according to the “fluid-mosaic model” (Singer and Nicolson 1972) forming the boundary of extra- vs. intracellular compartments. According to the results of state-of-the-art biophysical investigation methods, PMs can produce extremely diverse deviations from a smooth surface in many cell types. Among them are elongated or spherical protuberances, long prominences, deep valleys, buds, blebs, flat invaginations and other membrane structures that have been linked to specific functions in their respective cell type (Sharonov et al. 2016; Jacobson et al. 2019; Kalappurakkal et al. 2020; Saltukoglu et al. 2023). These topological configurations at the PMs are what we define as membrane landscapes (MLs).

Importantly, the transfer of MLs and organelles from mother to daughter cells and from gametes to the zygote occurs during cell division and cell fusion, respectively. Thereby a complete set of so-called “unit membranes” specific for the cell type, including PMs, ER, mitochondria, peroxisomes, etc., are passed from the “donor” to the “acceptor cells”. All other membrane systems, including the Golgi apparatus, secretory vesicles (Jamieson and Palade 1966; Taylor et al. 2023) and lipid droplets (Jackson 2019; Zadoorian et al. 2023), are derived from the ER through budding and fusion of small vesicles along the so-called “Palade” or “secretory pathway”. This had been first described over 60 years ago (Jamieson and Palade 1967). “Unit membranes” with unique distribution, topologies and orientation of their constituting components, i.e., organelles and MLs, are thus not replicated *de novo* by “self-assembly” (as holds true for ribonucleic acid proteins, such as ribosomes, SRP or multi-protein complexes) or along the “secretory pathway” (as holds true for the Golgi apparatus and secretory vesicles). Rather, sets of “unit membranes” grow by the incorporation of proteinaceous and lipidic constituents into pre-existing structures which have originally been derived from the mother cell or gamete upon transfer to the daughter cell or zygote, respectively. For the replication of the “unit membranes,” sophisticated mechanisms involving complex machineries of protein channels and transporters, specific for each “unit membrane,” have been developed. They ensure in bacteria as well as in lower (yeast) and higher eukaryotes the appropriate targeting and incorporation of individual protein constituents into the correct membrane precursors. This occurs after protein synthesis, i.e., in post-translational mode for mitochondria (Harmey and Neupert 1979; Nelson and Schatz 1979), a minority of ER (Müller and Zimmermann 1987; Juanes et al. 2008) as well as some bacterial proteins (Zimmermann and Wickner 1983) and all GPI-APs (Ast et al. 2013), or through co-translational processes for the majority of ER and secretory proteins (Milstein et al. 1972; Blobel and Dobberstein 1975), and some bacterial proteins (Müller and Blobel 1984). Thereby, cycles of replication of sets of “unit membranes” including MLs are completed, relying on specific and complex molecular machineries of membrane insertion, among them on signal recognition particle (SRP)- / SRP-receptor- and Sect. 62-dependent or independent, co- or post-translational modes (Andrews et al. 1989; Meacock et al. 2000; Jadhav et al. 2015) as well as mixed co- and post-translational ones (Steinberg et al. 2020; Jung et al. 2021). Importantly, those machineries must be transferred during cell division to ensure the propagation of MLs in the daughter cells. Thus, even completely synthesized (i.e., small proteinaceous or GPI-anchored) membrane protein precursors (so-called GET proteins) do not underly self-assembly into their organelles and MLs, as originally described by the membrane trigger (Wickner 1980) and the helical hairpin hypotheses (Engelman and Steitz 1981) proposed more than 40 years ago. Rather biogenesis of organelles and MLs depends on proteinaceous cytosolic and / or membranous unfolding and / or insertion mechanisms consisting of chaperons, such as HSP70 (Shan 2023), SRP and Sect. 62 (Reithinger et al. 2013), and channel or gating components, such as TRC40, Snd1-3 (Aviram et al. 2016) and Sect. 61 (Haßdenteufel et al. 2019). Instead of individual constituents being spontaneously drawn to each other, organelles and MLs obey the rules of self-organizing systems. Those do propagate by their transfer from mother to daughter cells or organisms along the continuum of the soma and germ cell line, respectively, following the assembly of novel constituents into the corresponding pre-existing structures. This has also been emphasized in the theory of biological systems with the definition of a vital organism as a hierarchy of open systems, which succeeds to keep its form based on the continuous change of its substances in accordance with the systemic conditions (von Bertalanffy 1949). Subsequently, the self-referential, structurally coupled and autopoietic characteristics of systems theory have been emphasized (Varela 1979; Varela and Maturana 1987).

During recent years, novel mechanisms have been identified that lead to the transfer of MLs between somatic cells and possibly also from somatic to germline cells. In a case of lower complexity, GPI-APs, together with other membrane proteins and (lyso)phospholipids, are released from the surface of donor cells constitutively or in response to specific (patho)physiological stimuli or environmental factors (Müller and Müller 2023b, c), packaged into specific micelle-like structures (Müller et al. 2020). These complexes are then taken up by neighboring or distant acceptor cells and finally incorporated into their PMs (Müller et al. 2021a, b), where they may contribute to the formation of new MLs. As a consequence, a new phenotype is induced in those acceptor cells, e.g., through stimulation of lipid and glycogen synthesis in adipose and blood cells, respectively. This phenotype, which has not yet been manifested in the acceptor cells before transfer of the micelle-like GPI-AP complexes has been prevalent only in the donor cells (Müller and Müller 2022, 2023a).

Another non-vesicular mechanism for the intercellular transfer of MLs is the direct transfer of membrane proteins and phospholipids of a specific area from the PMs of the donor cell to the PMs of the acceptor cell *via* direct cell-to-cell contact (Bouma et al. 1977; Huestis and Newton 1986). This process, described for the first time more than 40 years ago as “trogocytosis”, is accompanied by the loss of MLs and its specific functions in the donor cell and the gain of MLs in parallel with those functions by the acceptor cell (Miyake and Karasuyama 2021; Hertz et al. 2023).

Finally, vesicular mechanisms responsible for the transfer of membrane proteins, including GPI-APs, and phospholipids, and their assemblies into MLs, and also of cytoplasmic constituents, such as soluble proteins, DNA and RNA, from donor to acceptor cells in a wide variety of organisms, have attracted great attention (Clemmens and Lambert 2018; Couch et al. 2021). A distinction can be made between the transfer of MLs by extracellular vesicles (EVs), i.e., microvesicles (Chargaff 1948; Crawford 1971; Trams et al. 1981; Johnstone 2005; Hargett and Bauer 2013; Cocucci and Meldolesi 2015) and exosomes (Müller et al. 2011; Müller 2012; Jeppesen et al. 2019; Mathieu et al. 2019; Raposo and Stahl 2019). There is also experimental evidence available for the possibility of the transfer of specific types of EVs (epididymosomes, prostasomes, uterosomes) from somatic cells to germline cells / gametes (Griffiths et al. 2008; Siciliano et al. 2008; Yanez-Mo 2015; Kusama et al. 2018; Kurian and Modi 2019; Nakamura et al. 2020; Godakumara et al. 2022). These vesicular mechanisms, especially the operation of exosomes, have so far been considered in most (Jeppesen et al. 2019; Mathieu et al. 2019; Raposo and Stahl 2019), albeit not in all cases (Valadi et al. 2007) as sophisticated modes of intercellular communication and signal transduction. Transfer of genetic or non-DNA matter of inheritance, more precisely of DNA (vesicular lumen) in concert with MLs (vesicular membranes), between somatic cells (Hargett and Bauer 2013; Jeppesen et al. 2019; Raposo and Stahl 2019) or between somatic and germline cells or zygotes (Griffiths et al. 2008; Kusama et al. 2018; Kurian and Modi 2019; Godakumara et al. 2022) has been considered as putative function of EVs only to a minor degree so far (see Suppl. Figure 1 for a model of the non-vesicular and vesicular modes of the transfer of GPI-APs). Both the non-vesicular and the vesicular modes of transfer of MLs represent possible molecular mechanisms of the inheritance of acquired features (see Suppl. Figure 2).

As early as in the 1930s till present times, several findings have been published that can only be adequately explained by the inheritance of acquired traits by non-genetic mechanisms (e.g., Hadorn 1932; Goldschmidt 1934; Michaelis 1948; von Wettstein 1946; Sonneborn 1949; Beisson and Sonneborn 1965; for a review see Barthelmess 1952; Nanney 1981; Sapp 1987; Harwood 1993; Jablonka and Lamb 1995; Preer 2006; Beisson 2008; Day and Bonduriansky 2011; Bonduriansky 2012). Vice versa, certain “like-from-like” phenomena between parents and their offspring cannot be explained adequately by genetic inheritance (e.g., see Agrawal 2014, 2017). However, it was only with the methodological and technological advances of recent decades that the molecular mechanisms behind non-genetic inheritance started to be understood (Landman 2022). Interestingly, some of these mechanisms cannot be traced back to epigenetic processes. With the discovery of EVs harboring nucleic acids (see above), free nucleic acids circulating in body fluids (Yakubov et al. 2002; Ziegler et al. 2002; Stroun and Anker 2005) and proteins capable of self-replication and “self-templating” (i.e., prions: Wickner et al. 2004; Shorter and Lindquist 2005), the possibility of the existence of non-genetic matter was again brought up for discussion (Liu 2009; Liu and Chen 2018). If these structures were granted the “status” of a non-DNA matter of inheritance, this interpretation would have long-reaching implications, not only for the practice of genetical, evolutionary and cell biological research, but also for the theory, sociology and history of science. So far, EVs and their intercellular transfer have been interpreted solely as an additional mechanism of the inheritance of DNA. In this respect, it does not matter whether the DNA is being wrapped as chromosomes, being encapsulated in vesicles or naked, being linear or circular, or being of bacterial or human origin. However, it matters that the EVs use an extracellular route, which is not based on cell division or fusion, i.e., the mode of transmission for genetic inheritance.

The replication of the DNA is not completely “autonomous”, though the structure of the double helix suggests so. Replication of the DNA strands critically depends on a complex sophisticated apparatus involving many proteins, other nucleic acids (e.g., primers) and even membranes which supports the semi-conservative mechanism of DNA replication. Thereby, each daughter cell receives one strand of the DNA double helix from the mother cell which undergoes completion to a double strand by the complementary strand before transfer. So, to a certain extent, there is continuity of matter that is transferred from cell to cell, from organism to organism (even if it becomes more and more “diluted” with generations). Importantly, replication of DNA apparently does not follow the rules of self-assembly, i.e., DNA is not generated “*de novo*”, but rather by the incorporation of newly synthesized constituent components, i.e., nucleotides, along an already existing “template”. The resulting double strand consists of the “template”, the “original” or “positive” and the “print”, “image” or “negative” derived thereof. The same holds true for the replication of PMs, organelles and MLs, with the corresponding machineries differing fundamentally from each other. They all grow by the incorporation of newly synthesized constituent components in pre-existing assemblies and structures.

Pre-existing PMs, MLs and organelles are indispensable prerequisites for providing shape to a new cell, driving its metabolism, and expressing its genetic information. As a template, such cellular assemblies and structures manage to trigger and direct the biogenesis of analogous structures in the daughter cells. Thus, an aspect of the cellular phenotype is inherited, which may be interpreted as the information for the ontogenetic re-construction of a particular structural variant. In eukaryotes, this “structural templating” guarantees the transfer of basal bodies, centrioles, cytoskeletal elements, mitochondria and chloroplasts in the course of either mitosis in organisms with asexual (protists, ciliates, amoebae, flagellates) or sexual (metazoa) reproduction, and thereby operates as matrix for replication (Jennings 1939; Beisson and Sonneborn 1965). From a historical point of view, the transfer of cytoplasmic and membranous formations and assemblies through “structural templating” in eukaryotic unicellular organisms has been known for quite a long time as cytoplasmic inheritance (for instance see, Grun 1976; Grimes 1982).

“Structural templating” or cytoplasmic inheritance does not follow *de novo* biosynthesis (Yaffe 1999; Moreira-Leite et al. 2001; Lockshon 2002; Cavalier-Smith 2004; Feldman et al. 2007; Beisson 2008; Shirokawa and Shimada 2016). The cell cortex, a cytoplasmic layer located at the inner leaflet of the PMs, which is often rich in cytoskeletal elements and important for the formation of extracellular bonds and interactions during cell movements, and the glycocalyx at the outer leaflet of the PMs and cell surfaces (also present in germ cells) represent a heritable temporal continuum – independent of DNA (Cremer 1985; Szathmáry 2000). This is the very reason why cells, irrespective of whether being of prokaryotic or eukaryotic origin, do not follow self-assembly and will not arise *de novo* upon construction of their genome with the aid of recombinant DNA technology. Nevertheless, some researchers consider the transfer of a complete chemically synthesized genome or chromosome into pre-existing viable bacterial or yeast cells as the *de novo* creation of new life (Gibson et al. 2010; Venetz et al. 2019; Schindler et al. 2023). In contrast, the “replication” or biosynthesis of individual cellular macromolecules, such as proteins, carbohydrates, or lipids, are generated *de novo*, i.e., they are built together from their constituent components (e.g., amino acids, fatty acids) with the help of DNA as the “instruction” or information for their assembly. This occurs either directly (proteins) by following the central dogma of molecular biology downstream to translation or indirectly (lipids, carbohydrates) by synthesis with the aid of the enzymes translated. This means that the constituents of a particular entity of a protein, carbohydrate, or lipid to be replicated, are not passed on to the copy of the specific protein, carbohydrate or lipid. So, there is no continuity of matter between the “old” and “new” entities of individual proteins, carbohydrates, and lipids. Temporal and spatial continuity could therefore be regarded as a criterion for a matter of inheritance, irrespective of whether being of DNA or non-DNA nature.

This criterion of formation of temporal and spatial continuity is fulfilled by MLs of PMs and organelles. These assemblies of membrane phospholipids and transmembrane and lipid-modified proteins, among them GPI-APs, are apparently kept together only by means of weak van der Waals, electrostatic and hydrophobic interactions, as well as hydrogen bonds. Admittedly, their potential to maintain and transfer structural information in a stable and long-lived fashion is limited. But it is precisely this imperfect mechanism of copying that may be responsible for hereditary structural changes in the typical eukaryotic “membranome”, i.e., the entirety of all cellular membrane systems consisting of at least 18 different members (Cavalier-Smith 2001; Gosh et al. 2008), in concert with ontogenic and phylogenic adaptations.

Taken together, biological membranes, in general, and MLs, in particular, do not obey the rules of *de novo* or self-assembly. They grow and replicate by the incorporation of the constituent components, proteins, and lipids, into pre-existing “mother” or “parental” organelles and MLs, which act as a type of template for “daughter” or “offspring” organelles and MLs, like the one DNA strand during replication of the DNA double helix. As with DNA, there is continuity of the matter of organelles and MLs. Parts of the “mother” or “parental” organelles and MLs become part of the successor structures, which are passed on to daughter cells from one generation to the next. The successful transfer is critically dependent on their correct incorporation into the organelles and MLs to be replicated. These incorporation processes, highly specific for membranes and MLs, involve complex molecular machineries, which has been intensively investigated during the last five decades. The only main difference between the replication of DNA and that of membranes and MLs is the “semi-conservative” and “disperse” mode of mechanism, respectively. This is manifested best in the base-pairing of DNA as well as in the recognition of protein constituents, among them GPI-APs, by cognate receptors at the targeted membranes and MLs among them cortical cytoskeletal elements (Sharonov et al. 2016; Jacobson et al. 2019; Kalappurakkal et al. 2020; Saltukoglu et al. 2023).

It is of crucial importance that MLs are susceptible to environmental factors, such as (oxidative) stress and mechanical distortion. MLs manage to respond through specific changes in their topology, orientation, configuration and assembly state. Those changes may affect the function of the cell and thus can cause changes on phenotypes (see Suppl. Figure 1 for the presentation of documented examples of MLs at PMs of lymphocytes). And these environmentally induced changes can be replicated by the incorporation of newly synthesized protein components into the altered MLs, which we termed “membrane environment landscapes” (MELs), just to stress the tight interaction of MLs and environmental factors. Taken together, the replication and transfer of MELs and their environmentally induced topological changes, which may be regarded as “non-genetic mutations”, could therefore represent a mechanism for the inheritance of acquired traits (see Suppl. Figure 2 for a model of environment-induced alterations of MELs and their intercellular transfer). Thus, MELs should be considered as dynamic and flexible structures, configurations and assemblies. They are capable to adopt different orientations and topologies of defined structural re-arrangements.

**Fig. 3:**
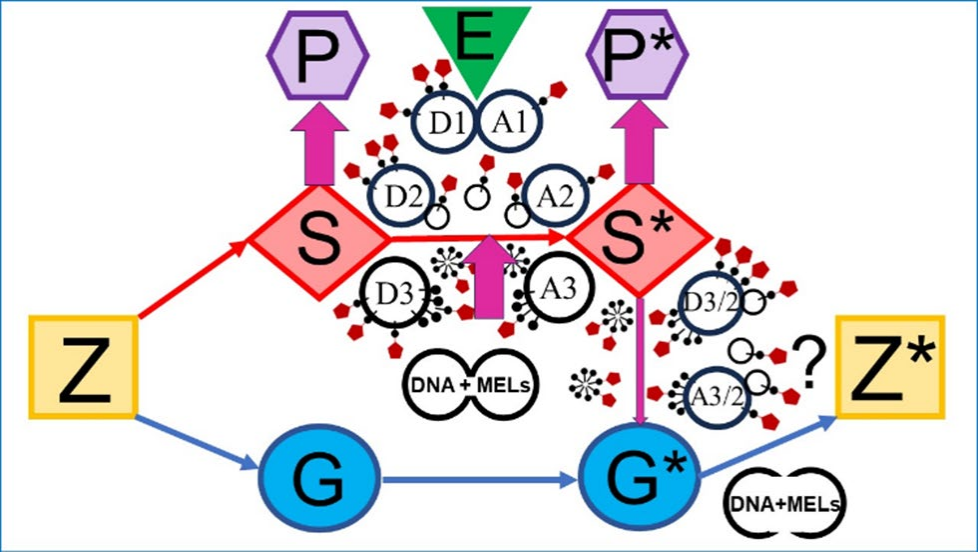
“Poly-matter network” conception. The DNA- / environment-centric“ conception (see Fig. 1) has been supplemented with the various extracellular modes of the transfer of MELs, from S to S* and S* to G*, involving either direct contact between donor cells D1 and acceptor cells A1 (trogocytosis) or the budding from donor cells D2 and fusion with / uptake by acceptor cells A2 of EVs or the release of micelle-like GPI-AP complexes from donor cells D3 and insertion into acceptor cells A3. For reasons of simplification, MELs are depicted here only as GPI-APs, but typically represent assemblies of transmembrane proteins, peripheral proteins, GPI-APs, prion-like proteins, and intrinsically disordered proteins of specific topology at PMs of S and, after transfer, also of S* and G*. Thus, extracellular transfer of MELs is assumed to occur in addition to the intercellular transfer of MELs and of other non-DNA matter along with PMs and organelles from S to S* in course of cell division. Transfer of DNA matter from S to S*, G to G* and parental G* to offspring Z* in course of cell division and fusion, respectively, and the operation of epigenetic mechanisms are fully acknowledged by the “poly-matter network” conception. However, at variance with the “holistic” view (see below), the necessity to indicate the need of continuous maintenance of E from generation to generation for a causal explanation of the reliable recurrence of P* in organisms constituted by S* is declined for the sake of simplification

In conclusion, various (sub)cellular structures and materials, including organelles, MELs, EVs and GPI-AP complexes, as well as their surroundings and environmental factors, are all actors in a “poly-matter network” of inheritance (Fig. 3). Importantly, the distinction between DNA and non-DNA matter of inheritance become visible and stabilized in the course of their agential separation, by using distinct methods of observation and production, such as centrifuges, gel chambers, microscopes, and the use of distinct model organisms.

In comparison, the “DNA- / epigenetics-centric” conception of inheritance (see above) provides a rather narrowed, i.e., “one-dimensional” explanation for the transgenerational effects of the environment on organisms and bodies. It predominantly relies on the exogenous control of the expression of certain genes, i.e., of the amount of their RNA and protein products. In contrast, the “poly-matter network” conception of inheritance bypasses this apparent narrowing and opens the multi-dimensional space of MELs to the input of environmental factors. These environmental factors may induce, either alone or in concert, (re-)configurations of the topography of MELs and organelles, leading to (re-)organized protuberances, valleys, blebs, invaginations etc., independent of whether being exposed at the surface (PMs) or hidden in the depth of the cytoplasm (at the nucleus, ER, Golgi, mitochondria). Thus, environmental factors produce inscriptions, spurs, marks in the MELs and organelles and thereby imprint their complexity / multi-dimensionality into non-DNA heritable matter.

In his recent book, Alfredo Martinez Arias (Martinez Aria 2023) substituted the “blueprint” analogy often applied in the context of DNA matter by a “hardware store catalog” image. He argues that the genetic information of an organism must be interpreted as the catalog used by the individual cell to order the building plans for its constituting proteins. In contrast to the common view for the role of genes in the biogenesis of (sub)cellular structures, Martinez Arias states that genes are used by cells to produce the building blocks for the formation of the three-dimensional structure of any complex trait, but that genes per se do not offer a plan for its organization in space and time. In the present manuscript, we expand this view about the inheritance of information for the synthesis of proteins to include the role of MLs as self-organizing non-DNA matter that harbors information for the topological and functional arrangement of their constituent polypeptides.

## Conclusions

In this study, we proposed a “poly-matter network” model of trait inheritance, in which not only DNA and its replication and transfer, but also the transfer of *all* other non-DNA materials and their effects account for the reliable recurrence of phenotypic traits across generations of cells and organisms (see Fig. 3). Those transferred non-DNA materials to be included in causal explanations encompass self-organizing assemblies of proteins and lipids, such as membranes, organelles, MELs, as well as environmental factors, such as mechanical stress, gravity force, food ingredients, UV-light etc. We suggest that non-DNA matter together with environmental factors, which may exert topological alterations on MLs, must be considered in causal explanations of inheritance.

One of the basic questions for future Science and Technological Studies on biological inheritance can therefore be outlined as follows: Why has the phenomenon of the inheritance of non-DNA materials been relegated to the realm of fable or branded as wishful thinking for about the last 75 years? In fact, the major sets of genetic and molecular biological data raised in the 20th century has been interpreted almost entirely as DNA being the only matter or information that is passed on “vertically” along the germline, i.e., from primordial cells to gametes. The sole emphasis in the DNA-centric model of inheritance has led to the following consequences:

(i) Exclusion of environmental factors that may have inscribed into the “book of life” and thereby mediate the inheritance of acquired traits.
(ii) Exclusion of the “horizontal flow” of material or information from mother to daughter cells during cell division (asexual reproduction) as well as from parental somatic cells *via* gametes to zygotes (sexual reproduction).
(iii) Changes in the development of the offspring being considered feasible only by (a) recombination (meiosis) after fertilization, (b) random mutations in the DNA of the parental germline, (c) random mutations in the DNA of the somatic cells of the offspring, and (d) interactions between genes and environmental factors during development.

The “DNA- / epigenetics- / environment-centric” conception is still being considered as a sufficient explanation for the inheritance of traits. Under this conception, the fact that materials other than DNA are necessary for the development of organisms, that DNA is not causally sufficient to explain all heritable variation of phenotypic traits, and that membranes, in general, and organelles, PMs, and their MELs in particular, do not arise *de novo* is taken for granted but regarded as trivial and self-evident. Albeit the merits of the DNA-centric conceptions of inheritance regarding the prediction of the phenotype and its variation are highly acknowledged by the authors, these may lead to underestimation of the potential role of horizontal and vertical transfer of non-DNA matter, including its replication and transformation into phenotype by non-genetic mechanisms, such as *via* EVs and micelle-like (GPI-AP) complexes. Therefore, we would like to present a broader perspective that has the potential to expand our understanding of the inheritance of innate and acquired biological features, with major implications for the study of phenotypic variation, including differences in (patho)physiological responses, and molecular explanations for the development of common complex diseases. The latter have already been demonstrated for the pathogenesis of type II diabetes, with the involvement of EVs, micelle-like complexes and GPI-APs, in shifting the burden of lipid loading from small to large adipocytes within the same or distinct tissue depots in heritable fashion and under regulation of antidiabetic sulfonylurea drugs (Müller and Müller 2024).

Last but not least, the willingness to study the proposed “poly-matter network” in combination with the underlying molecular mechanisms and to acknowledge an adequate differentiation from the former DNA- / epigenetics- / environment-centric” conceptions of inheritance may contribute to novel valuable insights about how novel species and traits might have originated in the past and could be generated in the future. Admittedly, the number of examples for which the operation of the “poly-matter network” had unequivocally been shown has remained rather limited so far. Nevertheless, we are convinced that this conception deserves more intense analysis in case studies, in general, and under natural conditions, in particular. Identification of the molecular mechanisms of the replication (by self-organization), transfer (from donor to acceptor organisms) and expression (of phenotypes) as well as of their variations at the individual and population levels may facilitate our understanding and add new views about the natural processes in the production of novel forms and shapes out of the existing matter. Moreover, in addition to putative benefits for a deeper knowledge of the emergence of diversity during evolution, the corresponding engagement should be helpful for the construction of new traits and organisms in future efforts of synthetic biology.

## Electronic supplementary material

Below is the link to the electronic supplementary material.

Supplementary Material 1

## Data Availability

No datasets were generated or analysed during the current study.
