# Supplementary material for: A “poly-matter network” conception of biological inheritance
Source: Genetica. 2024 Oct 19;152(4-6):211–30. doi: 10.1007/s10709-024-00216-1 (PMC11541361; doi:10.1007/s10709-024-00216-1)
Supplement: Supplementary file 1 — Supplementary Material 1 [file 10709_2024_216_MOESM1_ESM.pdf]

Supplementary Figure 1

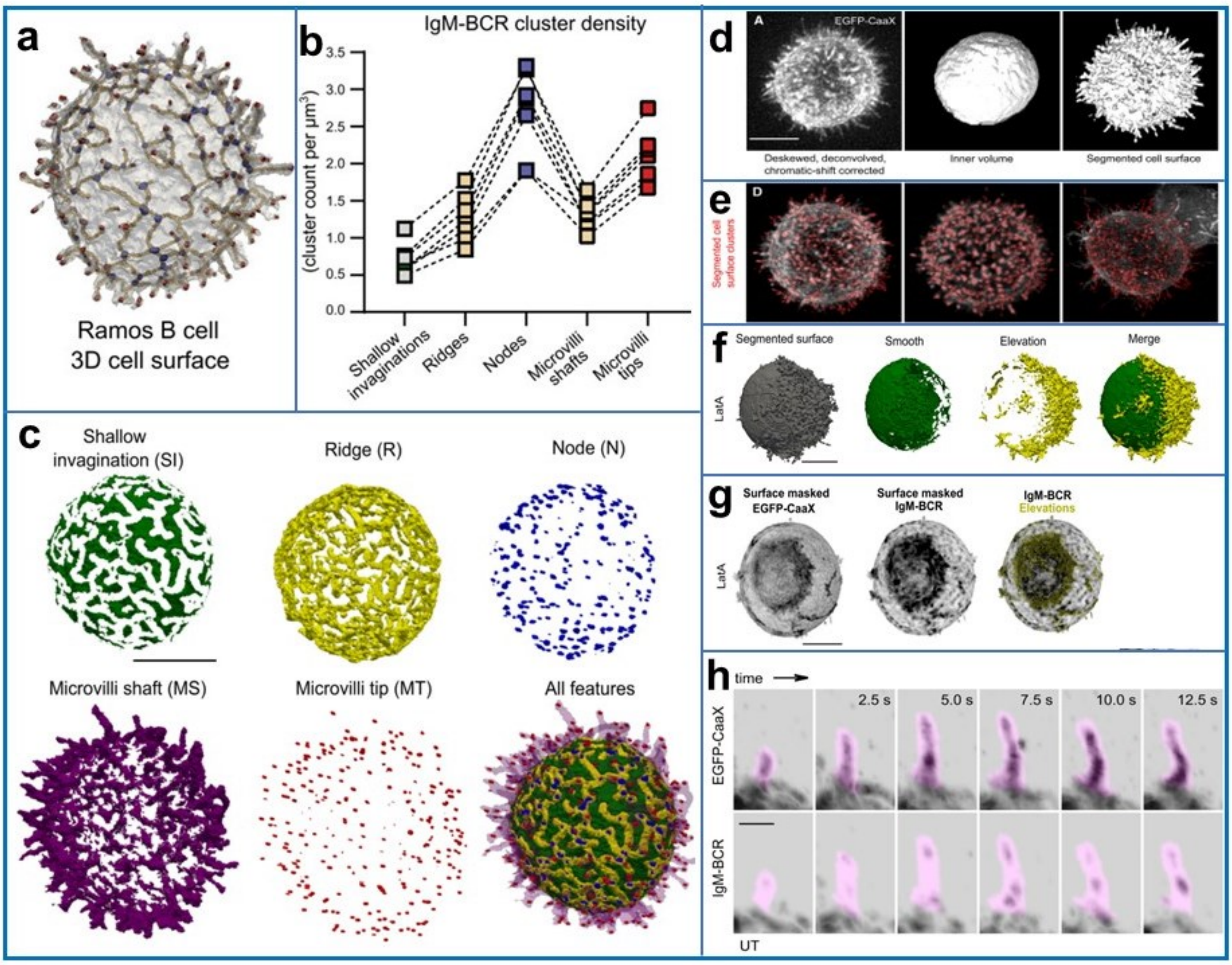

Identification of MLs at the PMs of Ramos B lymphocytes using a combination of volumetric high-speed microscopy with custom 4D image analysis demonstrating their constitution by elevated topographical features which are organized in a dynamic network-like pattern (adapted with modifications from Saltukoglu and coworkers [Saltukoglu et al. 2023] with permission of the authors). (**a**) Transmembrane proteins, such as IgM-class B cell antigen receptor (IgM-BCR), form clusters that are coupled to the topographical 3D features (ridges and microvilli) at the PMs. The skeletonized ridge network including the microvilli protrusions was superimposed on the mesh representation of the PMs. The ridges, their connecting nodes and the microvillar tips are indicated by yellow, blue, and red color, respectively. (**b**) The cluster density of IgM-BCR within different topographical features were quantitatively evaluated as cluster count per surface feature volume. (**c**) For visualization of the topographical features at the B lymphocyte PMs, the cell body was separated from the microvillar protrusions using mathematical morphology, with computation on the cell body of the ridge network (R; yellow) and annotation of its nodes (N; blue), the remaining PM area as shallow invaginations (SI; dark green) and the individual microvilli as separated into their shafts (MS; purple) and tip (MT; red). In addition, all featured were combined in a single image (scale bar = 5 µM). (**d**) For visualization of the B lymphocyte PMs, a 3D image of the distribution of the lipid-modified (farnesylated) green fluorescent protein EGFP-CaaX upon its expression in a Ramos B cell, which had then be embedded in Matrigel (left panel), a segmentation of the inner dome (middle panel) using the author’s own script based on Watershed segmentation and a segmentation of the

PMs (right panel) using an nnU-Net model, which had been trained by the authors based on their self-generated ground truth, were prepared (scale bar = 5 µM). (**e**) Cluster masks generated by a custom cluster detection algorithm based on local contrast. (**f**) For visualization of clustering of IgM-BCR at PM elevations induced by Latrunculin A (causing depolymerization of the cortical actin cytoskeleton), fixed and permeabilized Ramos B cells expressing EGFP-CaaX and IgM-BCR were stained with Phalloidin-Alexa555 and then imaged. Latrunculin A-treated cells display disorganized and polarized elevations (yellow) which are morphologically clearly separated from smooth regions (green) (scale bar = 5 µM). (**g**) Surface-masked EGFP-CaaX and IgM-BCR signals and overlay of the segmented elevations (yellow) with the surface-masked IgM-BCR signal in Latrunculin A-treated cells (scale bar = 5 µM). (**h**) For imaging of IgM-BCR cluster dynamics that is linked to the ridge network dynamics, time-courses of EGFP-CaaX and IgM-BCR intensity were prepared for an elongating microvillus of an untreated Ramos B cell (scale bar = 1 µM).

Supplementary Figure 2

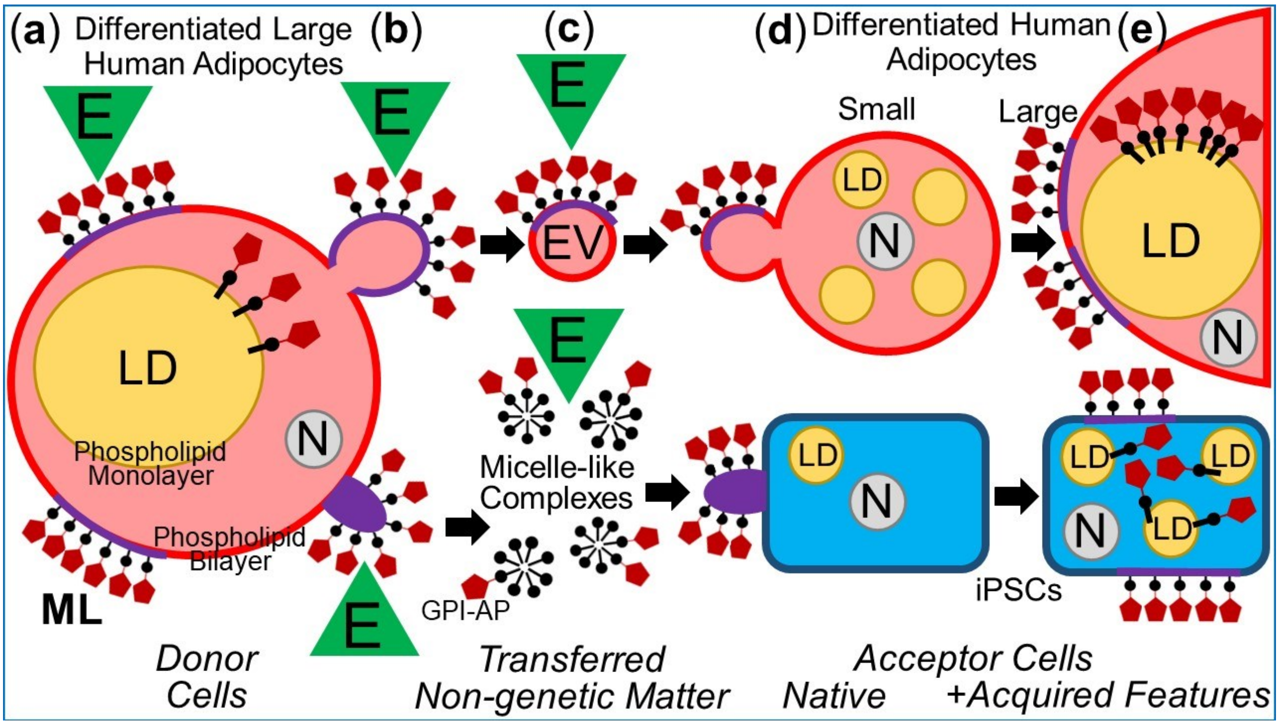

Model for the extracellular transfer of non-genetic matter, such as MLs (or MELs following environment action E), from donor to acceptor cells (adapted with modifications from Müller and Müller [2023c] with permission of mdpi press, Basel). (**a**) Differentiated large human adipocytes as donor cells for non-DNA matter express considerable amounts of GPI-APs at the outer leaflet of the PMs with their GPI anchor inserted into the outer leaflet of the phospholipid bilayer (Ferguson and Cross 1985; Müller et al. 1994) and concentration within MLs (or MELs following environment action E), which are constituted by (glyco / sphingo)phospholipids harboring saturated fatty acids, cholesterol, transmembrane proteins, peripheral proteins, intrinsically disordered proteins, prion proteins and components of the intracellular cytoskeleton (Müller 2002; Saha et al. 2016). Environmental actions (E), such as mechanical stress, may affect the composition and / or configuration of lipid droplets (LDs) (Caseli et al. 2008; Müller et al. 2008a). In addition, GPI-APs are located at the cytoplasmic face of LDs through insertion of their GPI anchor into the surrounding phospholipid monolayer, among them the 5’-nucleotidase CD73 and the cAMP-binding ectoprotein (Müller et al. 1992)

and phosphodiesterase Gce1 (Müller et al. 2008b), that in concert coordinate lipid synthesis and lipolysis through control of the cAMP concentration at the immediate surface area of the LDs (Müller et al. 2008c). (**b**) The donor adipocytes release into the tissue bed or circulation EVs by budding of the PMs (microvesicles) or by exocytosis (exosomes, not depicted here) and / or micelle-like GPI-AP complexes by local detachment of the PM outer leaflet from the bilayer, that are all equipped with MLs (or MELs following environment action E). Release happens at the basal state but becomes stimulated considerably upon challenge of the donor adipocytes by E, such as oxidative stress (Müller et al. 2020a). (**c**) During the transfer of the MLs (or MELs following environment action E), such as EVs and micelle-like GPI-AP complexes, from donor to acceptor cells, E, such as blood flow and pressure, may affect the composition and / or configuration of the MLs (Müller et al. 2020b). (**d**) MLs (or MELs following environment action E), such as EVs and micelle-like GPI-AP complexes, fuse with the phospholipid bilayer and its outer leaflet, respectively, of the PMs of differentiated small human adipocytes or iPSCs as native acceptor cells (Müller et al. 2021). (**e**) The proper integration and distribution of the MLs (or MELs following environment action E) from EVs and micelle-like GPI-AP complexes along the PMs of the native acceptor cells in concert with the subsequent translocation of GPI-APs from the PMs to the surface of LDs and insertion into their phospholipid monolayer ultimately results in upregulation of lipid synthesis and LD biogenesis in the acceptor cells as a consequence of proper regulation of cAMP metabolism at the LD surface area (Müller 2011; Müller and Müller 2022, 2023; Müller et al. 2008b). In conclusion, the transfer of the non-DNA matter MLs (or MELs following environment action E), from differentiated donor to native acceptor cells via EVs and micelle-like GPI-AP complexes leads to their conversion into acceptor cells with newly acquired features, here enhanced lipid synthesis and LD biogenesis. GPI-AP(s), glycosylphosphatidylinositol-anchored protein(s); LDs, lipid droplet(s); N, nucleus; M(E)L(s), membrane (environment) landscape(s) (only GPI-APs are depicted as typical representative of all proteinaceous components, pink color indicates the typical set of phospholipids); E, environmental actors; EVs, extracellular vesicle(s); iPSC(s), induced pluripotent stem cell(s); PMs, plasma membranes.

Supplementary References

Caseli L, Oliveira RG, Masui DC, Furriel RP, Leone FA, Zaniquelli ME, Orbulescu J, Leblanc RM (2008) Rat osseous plate alkaline phosphatase as Langmuir monolayer – an infrared study at the air-water interface. J. Coll. Interf. Sci 320:476-482.

Ferguson MAJ, Cross GAM (1985) Trypanosoma brucei variant surface glycoprotein has a sn-1,2-dimyristyl glycerol membrane anchor at its COOH terminus. J Biol Chem 260:4963-4968.

Müller G (2002) Dynamics of plasma membrane microdomains and cross-talk to the insulin signaling cascade. FEBS Lett 531:81-87.

Müller G (2011) Let's shift lipid burden. Eur J Pharmacol 656:1-4.

Müller GA, Müller TD (2022) Biological role of the intercellular transfer of glycosylphosphatidylinositol-anchored proteins: Stimulation of lipid and glycogen synthesis. Int J Mol Sci 23:7418.

Müller GA, Müller TD (2023) Transfer of proteins from cultured human adipose to blood cells and induction of anabolic phenotype are controlled by serum, insulin and sulfonylurea drugs. Int J Mol Sci 24:4825.

Müller G, Over S, Wied S, Frick W (2008a) Association of (c)AMP-degrading glycosylphosphatidylinositol proteins with lipid droplets is induced by palmitate, H_2O_2 and the sulfonylurea drug, glimepiride, in rat adipocytes. Biochemistry 47:12774-12787.

Müller G, Schubert K, Fiedler F, Bandlow W (1992) The cAMP-binding ectoprotein from Saccharomyces cerevisiae is membrane-anchored by glycosylphosphatidylinositol. J Biol Chem 267:25337-25346.

Müller GA, Tschöp MH, Müller TD (2020b) Upregulated phospholipase D activity toward glycosylphosphatidylinositol-anchored proteins in micelle-like serum complexes in metabolically deranged rats and humans. Am J Physiol: Endocrinol Metabol 318:E462-E479.

Müller GA, Tschöp MH, Müller TD (2021) Chip-based sensing of the intercellular transfer of cell surface proteins: Regulation by the metabolic state. Biomedicines 9:1452.

Müller GA, Ussar S, Tschöp MH, Müller TD (2020a) Age-dependent membrane release and degradation of full-length glycosylphosphatiylinositol-anchored proteins in rats. Mech. Age. Develop 190:111307.

Müller G, Wetekam E-M, Jung C, Bandlow W (1994) Membrane association of lipoprotein lipase and a cAMP-binding ectoprotein in rat adipocytes. Biochemistry 83:2115-2125.

Müller G, Wied S, Jung C, Over S (2008b) Translocation of glycosylphosphatidylinositol-anchored proteins to lipid droplets and inhibition of lipolysis in rat adipocytes is mediated by reactive oxygen species. Br J Pharmacol 154:901-913.

Müller G, Wied S, Straub J, Jung C (2008c) Coordinated regulation of esterification and lipolysis by palmitate, H_2O_2 and the antidiabetic sulfonylurea drug, glimepiride, in rat adipocytes. Eur J Pharmacol 597:6-18.

Saha S, Anilkumar AA, Mayor S (2016) GPI-anchored protein organization and dynamics at the cell surface. J Lipid Res 57:159-175.

Saltukoglu D, Özdemir B, Holtmannspötter M, Reski R, Piehler J, Kurre R, Reth M (2023) Plasma membrane topography governs the 3D dynamic localization of IgM B cell antigen receptor clusters. EMBO J:e112030.
